# RELATIONSHIP BETWEEN METABOLIC HEALTH AND 5 EPIGENETIC CLOCKS IN THE BERLIN AGING STUDY II (BASE-II)

**DOI:** 10.1093/geroni/igac059.009

**Published:** 2022-12-20

**Authors:** Ilja Demuth, Sandra Düzel, Valentin Vetter, Johanna Drewelies, Yasmine Sommerer, Elisabeth Steinhagen-Thiessen, Denis Gerstorf, Lars Bertram

**Affiliations:** Charité Universitätsmedizin Berlin, Berlin, Berlin, Germany; Max Planck Institute for Human Development, Berlin, Berlin, Germany; Charité - Universitätsmedizin Berlin, Berlin, Berlin, Germany; Humboldt University Berlin, Berlin, Berlin, Germany; University of Lübeck, Lübeck, Schleswig-Holstein, Germany; Charité - Universitätsmedizin Berlin, Berlin, Berlin, Germany; Humboldt Universität zu Berlin, Berlin, Berlin, Germany; University of Lübeck, Lübeck, Schleswig-Holstein, Germany

## Abstract

The diagnosis of metabolic syndrome (MetS) relies on cut-off values for five cardiovascular risk factors, three of which must be met to make the diagnosis. While this approach has certain advantages in the clinical setting, it only allows a binary classification relying on cut-off values. Thus, individuals receiving the same MetS diagnosis may differ in terms of disease severity. We have recently developed a continuous measure of the metabolic load (MetL) that was calculated as a latent factor from indicators that are used to diagnose the MetS enabling a more differentiated assessment. DNA methylation age acceleration (DNAmAA) is a widely accepted biomarker of aging which can be derived from a variety of epigenetic clocks. However, it is still unclear which aspects of aging these biomarkers represent best. Aim of the current study was to evaluate whether and how the MetS and MetL relate to DNAmAA derived from five epigenetic clocks (7-CpG clock, Horvath’s clock, Hannum’s clock, PhenoAge, and GrimAge). MetS was diagnosed according to the AHA/IDF/NHLBI criteria (2009) and the latent MetL factor was extracted from a confirmatory factor analysis considering the same diagnostic parameters. DNAmAA estimated from PhenoAge and GrimAge were significantly associated with MetS (beta=0.036 and beta=0.063, both p<0.05) and MetL (both beta=0.009 and p<0.05) in regression analyses adjusted for age and gender including 1,100 participants from the follow-up assessments of the Berlin Aging Study II (BASE-II, mean age 75.6 ±3.8 years). The results will be discussed with respect to clinical relevance in the context of current literature.

